# “*I was told to live happily… without telling me how*”: Lived experiences of married adolescents, their spouses and families with a life skills intervention in rural Pakistan – A qualitative study

**DOI:** 10.1017/gmh.2026.10240

**Published:** 2026-06-09

**Authors:** Najia Atif, Syed Usman Hamdani, Zill-e- Huma, Maria Kanwal, Ahmreen Kaukab, Mahjabeen Tariq, Atif Rahman

**Affiliations:** 1 Human Development Research Foundation, Pakistan; 2 Global Institute of Human Development, Shifa Tameer-e-Millat University, Pakistan; 3 Department of Primary Care and Mental Health, University of Liverpool, Liverpool, UK

**Keywords:** life skills, adolescent marriage, parenting, Pakistan, qualitative research

## Abstract

Adolescent marriage and early childbearing remain common in rural Pakistan, yet few interventions support young couples transitioning into parenthood. This study explores the perceived impact and implementation of preparing for parenthood (PPP), a life skills intervention designed for married adolescents and their families. A qualitative design explored participants’ and delivery agents’ experiences of PPP. Data were collected through 12 in-depth interviews and 4 focus group discussions with married adolescent women, spouses, family members and programme trainers. Thematic analysis was used. PPP was perceived as relevant, acceptable and empowering. Participants reported improved core life skills; communication, emotional regulation, problem-solving and decision-making that enhanced their confidence and preparedness for parenting. Home-based delivery by trusted female trainers and recruitment via community health workers supported engagement in mobility-restrictive, patriarchal settings. Challenges included limited male participation, family gatekeeping and the burden of domestic work. Participants suggested adaptations such as mobile content, male facilitators and media-based delivery to overcome these barriers. This study highlights the value of life skills interventions in equipping married adolescent women with socioemotional competencies necessary for effective parenting. Home-based, culturally grounded models paired with flexible, gender-sensitive approaches can foster empowerment and improve outcomes in low-resource, gender-restrictive settings.

## Graphical abstract

**Figure:**
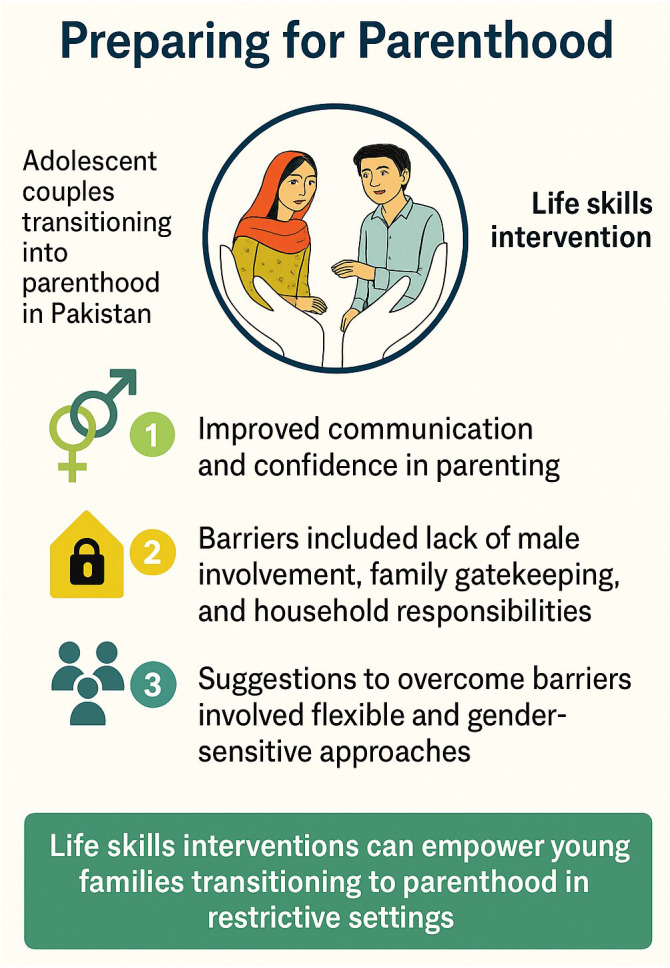

## Impact Statements

This study sheds light on the emotional and relational challenges faced by married adolescent women and demonstrates how a structured psychosocial support can strengthen communication, empathy and emotional resilience. The findings show that life skills interventions can play a critical role in improving adolescent mental health and wellbeing, particularly in settings with limited access to mental health services. By engaging husbands and other key family members, the intervention promoted stronger relationships and a greater understanding of emotional needs, extending benefits beyond individual participants. This research supports the integration of life skills and mental health components into adolescent and maternal health services in low-resource settings. It offers insights for policymakers and practitioners seeking culturally appropriate and scalable approaches to psychosocial support. Beyond the local context, the study contributes to global evidence by demonstrating how community-based, culturally grounded interventions can effectively address adolescent mental health needs in low- and middle-income countries.

## Introduction

Young people aged 15–24 make up 27% of the global population, yet their socioemotional well-being remains critically neglected, especially in low- and middle-income countries (LMICs) (Gore et al., 2011). In Pakistan, this gap is stark: 30% of the population (65.4 million) is aged 10–24, and nearly half (32.4 million) are not in education, employment, or training (UNICEF Pakistan, 2020). These challenges are compounded by the widespread practice of early marriage and childbearing. The median age at first marriage is 20.4 years for women and 25.9 years for men, with 30% of women married before 18 (NIPS, 2018). Contraceptive use is low – only 34% of married women aged 15–49 use modern methods, with access concentrated among the educated, urban and economically advantaged. Early marriage and limited contraception use lead to early childbearing: the median age at first birth is 22.8 years, and 13% of women give birth before the age of 18 (NIPS, 2018). These patterns are embedded within broader structural inequalities. Early marriage is not only a consequence of poverty, limited educational opportunities and youth unemployment, but also reinforces these conditions by curtailing girls’ education, restricting labour force participation and perpetuating intergenerational disadvantage. This bidirectional relationship has been documented across diverse global settings (Wahhaj, 2022).

Key life transitions, such as marriage, parenthood and financial responsibility, frequently occur with minimal preparation, increasing vulnerability to psychosocial distress and maladaptive parenting practices. Adolescent marriage, in particular, is significantly associated with controlling behaviour and intimate partner violence in Pakistan (Nasrullah et al., 2014). The UNICEF (2024), report highlights maternal conditions as leading contributors to disability-adjusted life years (DALYs) and mortality among adolescent girls. In a study conducted in rural Pakistan, young married women aged ≤20 are five times more likely to experience depression relative to their unmarried peers, attributed to abrupt role transitions, restricted autonomy and limited access to sexual and reproductive health services (Rahman et al., 2009).

These vulnerabilities are rooted in broader socio-cultural and economic factors. Early marriage in Pakistan is shaped by a complex interplay of these factors, notably entrenched patriarchal norms and traditional practices that often prioritise family honour over a girl’s well-being and education (Pourtaheri et al., 2024). Marriages are often arranged by families, with parents-in-law playing a central role in decision-making and in shaping young couples’ post-marital lives. While not all early marriages are forced, varying degrees of adolescent agency exist within these arrangements, often constrained by prevailing social norms.

Life skills interventions, targeting psychosocial, behavioural, cognitive and interpersonal competencies such as communication, emotional regulation, decision-making and problem-solving, have been shown to improve health and social outcomes among adolescents (WHO, 2003; Singla et al., 2020; Sherif et al., 2023). However, these interventions are predominantly school-based and often exclude issues linked to complex family dynamics and early marriages. This gap is particularly evident in LMICs, where life skills programmes are less systematically implemented and evaluated compared to high-income countries (Nasheeda et al., 2019). In Pakistan, where adolescent marriage is common, there remains a lack of targeted interventions addressing the unique challenges faced by married adolescents (Atif et al., 2021).

To address this gap, we developed a life skills intervention titled *Preparing for Parenthood (PPP)*, targeting married adolescent women, their spouses and families in rural Pakistan (Atif et al., 2021). Drawing on evidence from a systematic review (Singla et al., 2020) and strategies from the WHO-endorsed Thinking Healthy Programme (Rahman et al., 2008), this psychosocial intervention is based on the principles of Cognitive Behaviour Therapy (CBT) and focuses on three key areas essential to the wellbeing of newly married adolescent women: self-care, interpersonal relationships and preparation for parenthood. Designed for delivery by trained non-specialist female facilitators (programme trainers), the intervention includes 10 core and 10 booster home-based sessions on socioemotional life skills. Given the influential role of family members in shaping adolescents’ autonomy, health behaviours and parenting practices, the intervention engaged spouses and extended family to foster a supportive environment for behaviour change. This approach aimed to create conditions conducive to parenting readiness and enhanced parental efficacy. Further details on the intervention’s development and design are published elsewhere (Atif et al., 2021). The PPP was subsequently feasibility tested in a randomised controlled trial; detailed findings are reported elsewhere (Hamdani et al. *publication forthcoming*).

To complement the programme’s feasibility assessment, a qualitative evaluation was conducted within the trial to explore participants’ experiences and perceptions. This paper presents the experiences of married adolescent women aged 18–22, along with those of their spouses and families who participated in the programme. It highlights how the intervention’s grounding in local realities contributes to its relevance, acceptability and potential scalability. These qualitative findings offer insights into the design of inclusive, community-based models for supporting married adolescent women to enhance parenting efficacy in conservative, resource-limited settings where conventional service delivery systems often fall short.

## Methods

### Settings and participants

This qualitative study was nested within the feasibility randomised controlled trial of the PPP programme and included 60 married adolescent women (30 in each arm), aged between 18 and 22 years, who had been married for up to two years. The criterion of being married for up to two year was added to ensure that participants were newly married. The trial was conducted from May 2018 to April 2020 in rural Gujar Khan, a rural sub-district of Rawalpindi in Punjab, Pakistan, with an estimated population of 300,000. The area is predominantly agrarian, with most families living in joint households averaging 6.2 members. It faces high poverty (25% living on less than US$3.50/day), elevated fertility rates (3.8 births per woman) and low female literacy (below 45%). Approximately 95% of women are registered with Community Health Workers called Lady Health Workers (LHWs), and participants for the trial were recruited from their catchment areas. LHWs were asked to identify and provide a list of newly married adolescent women in their assigned areas who met the eligibility criteria.

To ensure the diversity within the sample for this study, a purposive sampling strategy was employed. Participants were selected from the intervention arm of the trial and included those who had either completed (i.e., received at least 10 out of the 20 sessions) or not completed (i.e., received fewer than 10 sessions) the intervention. Additional sampling criteria included the number of months married and the level of formal education. Thirteen eligible participants (8 completers and 5 non-completers) were approached by the research team via telephone. Of these, 8 completers and 2 non-completers expressed interest; 3 non-completers did not participate due to migration out of the study area (*n* = 2) or lack of interest (*n* = 1). Those who agreed were provided with an information sheet and consent form in person and given 24 h to consider participation. Interviews were conducted after informed consent was obtained.

### Data collection

Data were collected in Urdu (national language) through in-depth interviews (IDIs) and focus group discussions (FGDs) between December 2019 and February 2020 by two female research assistants. Both held bachelor’s degrees in social sciences, were employed by the host organisation and had prior experience in qualitative research. The research assistants were local, fluent in Urdu and had no prior relationship with the participants. Their shared gender and cultural background facilitated rapport; however, potential social desirability bias was mitigated through training in neutral interviewing and emphasis on confidentiality and voluntary participation. IDIs were conducted with married adolescent women and their spouses, while FGDs were held with the programme trainers and field coordinators, and participants’ family members. IDIs with completers were conducted until no new codes emerged indicating that the data saturation point was achieved.

Separate topic guides were developed in Urdu for each group; participants, families and trainers. These guides were pilot tested and refined based on feedback. Full English versions of the topic guides are available as Supplementary File 1. The guides explored key areas, including: experiences of participating in the programme, and facilitators and barriers to receiving or delivering the intervention along with recommendations for improving future programme delivery.

IDIs were conducted in participants’ homes and lasted between 30 and 60 minutes, while FGDs were conducted at the rural health centre and lasted between 90 and 120 minutes. A note-taker recorded observations during FGDs, which were later used during data analysis. Privacy was ensured during all interviews. Participants’ basic demographic information was recorded. All interviews were audio-recorded and transcribed in Urdu. During transcription, all data were anonymised. Transcripts were securely stored in locked filing cabinets at the study sites.

### Data analysis

The data were analysed using thematic analysis, informed by a primarily inductive approach that allowed themes to emerge from the data, alongside sensitising concepts derived from the intervention’s theoretical framework. This resulted in a hybrid inductive–deductive analytic approach.

Thematic analysis followed the six-phase approach outlined by Braun and Clarke (2006). It involved: (1) familiarisation with the data through repeated reading of transcripts; (2) generating initial codes systematically across the dataset; (3) searching for patterns to identify potential themes; (4) reviewing and refining themes to ensure coherence and relevance; (5) defining and naming themes; and (6) producing the final report with illustrative quotes to support each theme.

Data from each participant group were initially analysed separately to capture group-specific perspectives. Subsequently, we compared and integrated the findings to identify shared patterns in experiences related to the intervention’s acceptability, feasibility and perceived impact. This approach was adopted to provide a more holistic understanding of the intervention across its key stakeholders.

Reflexivity was an ongoing and integral component of the analytic process. Researchers engaged in regular critical reflection on how their disciplinary backgrounds, assumptions and positionality, particularly in relation to socioeconomic and cultural differences with participants may have influenced data interpretation. These reflections were facilitated through weekly supervisory meetings, where emerging codes and themes were discussed, challenged and refined.

To enhance the trustworthiness and credibility of the findings, transcripts were independently analysed by multiple researchers (*n* = 3), field notes were consulted and interpretations were discussed iteratively to ensure depth and consistency of analysis. These processes supported methodological rigour and helped mitigate unintended bias (Morse, 2015).

The study adhered to the Consolidated Criteria for Reporting Qualitative Research (COREQ) to ensure transparent reporting of the study design, participant selection, data collection and analysis. The complete COREQ list is provided as Supplementary File 2.

### The intervention

*Preparing for Parenthood (PPP)* is a manualised life-skills intervention for married adolescents, their spouses and families, delivered through home-based sessions of up to one hour over a period of 6 months. The programme includes an introductory session, followed by 10 core and 10 follow-up sessions, delivered weekly (see Table 1). These sessions focus on communication skills, problem-solving, relationship assessment, stress management, emotional regulation, identifying/eliciting affect and self-awareness. The intervention is delivered by programme trainers, who complete 35 hours of classroom-based instruction led by a senior trainer, with ongoing competency assessment through role-plays. Trainers who successfully complete the classroom component proceed to supervised field training, during which they deliver 10 core sessions to couples similar to the target population. Trainers received fortnightly supervision throughout the trial to review challenging cases, ensure competency in delivering the intervention and support their own wellbeing.

**Table 1.** Preparing for parenthood sessions Table 1. long description.

Session 0: Introduction to the program	
*Title of core sessions 1 to 10	Major focus of session
Session 1: Knowing and appreciating each other	Family support; communication skills
Session 2: Looking after yourself	Thought challenging; behavioural activation
Session 3: Looking after yourself and others around you	Thought challenging; behavioural activation
Session 4: Listening and understanding each other	Communication skills; awareness
Session 5: Communicating respectfully and positively	Communication skills: assertiveness
Session 6: Seeking and valuing each other’s opinion	Communication skills; decision making
Session 7: Preparing for parenthood: pre-pregnancy	Awareness and education of pre-pregnancy care; behavioural activation; problem solving
Session 8: Preparing for parenthood: during-pregnancy	Awareness and education of care during pregnancy; behavioural activation; problem solving
Session 9: Preparing for parenthood: post-pregnancy	Awareness and education of post pregnancy case; behavioural activation; problem solving
Session 10: Managing problems	Problem solving

*Note*: *Each core session has a follow-up session, delivered the following week, making a total of 20 sessions.

### Ethical approval

Ethical approval for the study was obtained from the institutional review board of the Human Development Research Foundation (HDRF), Pakistan. All participants gave written informed consent before participation in the study.

## Results

### Qualitative results

In total, 12 IDIs and 4 FGDs were conducted. 10 IDIs were conducted with female participants who were recruited in the intervention arm (*n* = 8 completers, *n* = 2 non-completers) and 2 IDIs with husbands. The participants’ ages ranged between 19 years and 22 years, years of schooling between 5 years and 12 years and most of them were married for less than a year (see Table 2 below). Each IDI lasted between 30 to 60 min, they were recorded and transcribed. Three FGDs were conducted: these included the participant and her family members (e.g., mother-in-law and sisters-in-law), with each FGD comprising 3 to 5 family members from the same family (see Table 3 below). One additional FGD was conducted with the programme trainers and the Field Coordinators (4 trainers and 3 field coordinators). Data analysis generated four themes with three to four subthemes grouped under each theme. These are presented in Table 4 along with their key findings. Quotes used to support sub-themes are attributed using pseudonyms. Details are given below.

**Table 2.** Demographic information of married women and their spouses – participants of IDI Table 2. long description.

Reference No	Pseudonym	Age	No of married years	Family structure	Years of schooling	No of children
*C1	Amina	21 years	1 year	Joint	10 years	Nil
C2	Sana	22 Years	1 year	Joint	12 Years	Nil
C3	Nadia	20 Years	11 Months	Joint	10 Years	Nil
C4	Zara	19 Years	1 Year	Joint	12 Years	Nil
C5	Fatima	19 years	10 Months	Joint	10 Years	Nil
C6	Ayesha	19 Years	10 Months	Joint	5 Years	Nil
C7	Sara	21 Years	1 Year	Joint	9 Years	1
C8	Saba	20 Years	2 Years	Joint	12 Years	Nil
*NC1	Nabila	20 Years	1 Year 3 Months	Joint	10 Years	Nil
NC2	Maira	20 years	2 years	joint	10 years	1
*HC1	Hassan	28 Years	10 Months	Joint	5^th^ Years	Nil
*HNC2	Ali	27 Years	2 Years	Joint	8 Years	1

*C = participants who completed the intervention (received ≥10 sessions).

*NC = non-completers participants (received >10 sessions).

*HC1 = husband of the participant who completed the intervention.

*HNC1 = husband of the non-completer participant.

**Table 3.** Participants of focus group discussions Table 3. long description.

References	Number of participants	Relationship to the study participants
F1	3	Family: Mother-in-law, sister-in-law, participant
F2	4	Family: Mother-in-law, sister-in-law x 2, participant
F3	5	Family: Mother-in-law, sister-in-law x 3, participant
T&F	7	Trainers and Field Coordinator

**Table 4.** Thematic table with key findings Table 4. long description.

Themes		Key finding
**1.0**	**Perception of the intervention**	Participants welcomed the intervention as a timely and relevant opportunity to gain life skills and prepare for parenting. They appreciated the flexible, home-based format that allowed family involvement and fit within their routines. Interactive techniques like storytelling, role-play and relatable materials enhanced engagement, emotional connection and practical application of learning. Participants reported actively applying the life skills learned, especially in self-care, communication and stress management.
1.1	*Expectations and motivation*	
1.2	*Satisfaction with delivery format*	
1.3	*Techniques and materials*	
1.4	*Ease of application of life skills*	
**2.0**	**Facilitators of intervention engagement and delivery**	Family support was critical for women’s engagement, with in-laws’ involvement enhancing acceptance and uptake of the intervention. Participants actively shared lessons with husbands and relatives, though concerns were raised about potential miscommunication. Trust in trainers, who were locally recruited and culturally attuned, played a central role in participants’ sustained involvement, with many describing them as approachable, respectful and emotionally supportive. Lastly, perception of the positive impact, such as improved overall emotional well-being and relationships, facilitated engagement. Many women felt better equipped for their parenting roles, with improved decision-making and shared responsibilities. Behaviour change was also noted by trainers and spouses.
2.1	*Family support and participation*	
2.2	*Sharing knowledge with husbands and in-laws*	
2.3	*Trust and rapport with trainers*	
2.4	*Perceived positive impact of the intervention*	
**3.0**	**Barriers to intervention engagement and delivery**	Husbands’ participation was limited due to work demands, cultural norms and in some cases, disinterest. Family resistance rooted in control, distrust of outsiders, or fear of disclosure led some women to withdraw or engage reluctantly. Competing domestic and agricultural responsibilities, alongside the perceived burden of multiple sessions, further constrained regular attendance and full participation. Internal barriers include low literacy, initial difficulty with certain techniques and lack of motivation, highlighting the need for continued support and culturally sensitive delivery.
3.1	*Limited husband participation*	
3.2	*Family control and resistance*	
3.3	*Competing responsibilities and perceived time burden*	
3.4	*Internal barriers*	
**4.0**	**Recommendations for programme improvement**	Participants suggested using digital platforms like WhatsApp, audio recordings, or televised content to better engage men who face barriers to in-person participation. Culturally appropriate male-led delivery channels were recommended to increase men’s involvement. To improve accessibility and efficiency, participants also proposed restructuring the programme by reducing session frequency and delivering content in group formats at community venues such as schools or health centres
4.1	*Leveraging technology to engage men*	
4.2	*Engaging men through male-focused delivery channels*	
4.3	*Reducing and restructuring sessions*	

#### Theme 1: Perceptions of the intervention

##### Subtheme 1.1: Expectations and motivation

Most participants expressed a desire to gain knowledge to deal with life challenges and improve communication within their families. A recently married participant shared: “*When I decided to attend the programme, I was expecting that our family would become happier… that we would better understand each other’s point of view, and that our communication would improve.*” (IDI-Zara, aged 19 years).

Participants expressed their aspirations to become good parents and welcomed the opportunity to receive the intervention. A young mother-to-be shared: “*Nobody asks you if you are ready to be a mother or not. If you get married, you are expected to have a baby within a year or so. I used to think, will I be a good mother? I appreciated it when I was told about this programme.*” (IDI-Fatima, aged 19 years).

While the majority showed some interest, a few were unsure about joining the programme. One participant expressed uncertainty about her participation, stating: “*My sister-in-law, who was about to get married, was very keen for me to join the programme; I agreed at her persuasion without being fully convinced.*” (IDI-Ayesha, aged 19 years).

##### Subtheme 1.2: Satisfaction with delivery format

Participants appreciated the programme’s structure, comprising 20 sessions: 10 core, 10 follow-up and an introductory session, delivered fortnightly at home over six months. Delivery of the sessions at home was particularly important given that all trial participants were living in joint family structures, where newly married women typically move into their husband’s parental home and reside with in-laws within hierarchical family systems that often limit autonomy. In this context, the home-based model was highly valued, as it facilitated comfort, flexibility, and involvement of family members:

“*There is nothing better than home, you can go in a separate room and talk. If it would have been outside the house, family members could not be involved………. sometimes I used to take a break* *(from the session) to give my father* *-in* *-law* *lunch… If I had to go somewhere, I might not have been able to attend.*” (IDI-Sana, aged 22 years).

Follow-up sessions were seen as especially beneficial for reinforcing learning, addressing challenges, and practicing life skills: “*Follow-up sessions… helped them to implement the life skill and master it by practicing it for two weeks.*” (FGD-Trainer).

##### Subtheme 1.3: Techniques and materials

Participants’ highlighted storytelling, group discussions and role-play as engaging and relatable. Storytelling was particularly appreciated for its emotional resonance and cultural relevance: “*The best thing was storytelling… we used to wait to receive the sessions.*” (FGD-Sister-in-law).

Participants were encouraged to practise life skills such as empathetic listening, problem-solving and assertiveness during their sessions. This enabled them to apply these skills in their day-to-day interactions: “*I told my husband about active listening, but he didn’t understand… then I did role play… and then he understood.*” (IDI-Fatima, aged 19 years).

Furthermore, the intervention materials were considered relevant and easy to understand, particularly because they reflected participants’ real-life experiences. As one wife explained: “*There was no story which I didn’t like, all stories were good. They really touched my heart because they were so similar to my situation. Rashda (character in the story) used to sit alone and cry… I used to do that too.*” (IDI-Sana, aged 22 years).

A husband expressed similar view, stating: “*This book is written about my life… My wife was against my wishes to go abroad, but after participating (her understanding and compassion for my needs enhanced and) she gave me permission.*” (IDI-Hassan, aged 28 years).

Almost all participants were able to understand Urdu, but they expressed feeling more comfortable communicating in Potohari (local dialect). This was reported by both participants and their trainers. Trainers observed that using the local dialects, along with contextually relevant examples, enhanced participants’ understanding and engagement: “*We used to deliver the session in Potohari… Participants used to (appreciate that and) say that we knew about these things (skills) but never realised how important they are.*” (FGD- Trainers).

##### Subtheme 1.4: Ease of application of life skills

The intervention covered eight core life skills: self-awareness, communication, assertiveness, decision-making, goal-setting, critical thinking, problem-solving and stress management. Most participants reported actively applying these skills, particularly in self-care, communication and stress regulation: “*I have learnt how to behave according to my circumstances, keep my thoughts positive… and share my concerns with others. Even if there’s no solution, I feel relieved after off-loading.*” (IDI-Sana, aged 22 years).

A woman who married at a very early age recalled: “*At the time of my marriage, I was very young and had no idea how to manage life. My parents were not well educated; they simply told me to live happily with my husband and in-laws, without telling me how to. In this programme, I learned many new things.*” Appreciating the pregnancy-related content, she further added: “*I was pregnant and I felt I can apply the information from the sessions to take better care of myself.*” (IDI-Ayesha, aged 19 years).

#### Theme 2: Facilitators to the intervention delivery

##### Sub-theme 2.1: Family support and participation

Family involvement was crucial in enabling women’s participation in the intervention. Gaining permission from key household members helped foster acceptance of the programme, A mother-in-law stated: “*I am glad that she (the trainer) approached me first, explained the programme, and asked for my permission. I told her she could come anytime.*” (FGD-Mother-in-law).

Involving in-laws also reduced misunderstandings and allowed them to benefit from the content: “*My mother-in-law and sister-in-law used to attend the sessions. If all the female members sit together and listen, they learn the good things too*” (IDI-Sana, aged 22 years).

A younger participant reflected on the collective nature of decision-making in joint families: “*These skills can only be applied if the whole family has knowledge about them. A wife cannot apply them alone without family support.*” (IDI-Saba, aged 20 years.

##### Subtheme 2.2: Sharing knowledge with husbands and in-Laws

Participants were encouraged to engage family members in homework activities and to discuss session content with significant family members who could not attend the session. Some successfully involved their husbands, even remotely: “*My husband is overseas… I shared everything with him over the phone. He was interested and we did the activities while on the call*” (FGD-Faiza, aged 21 years). Others used storytelling to convey lessons and influence behaviour: “*I told my husband Akbar’s story and advised him not to argue after listening to outside gossip… He said alright.*” (IDI-Nadia, aged 20 years).

However, one trainer expressed concern about the accuracy of information being relayed to others: “*We don’t know how faithfully the information is conveyed… it might be misunderstood or selectively shared.*” (FGD- Trainers).

##### Sub-theme 2.3: Trust and rapport with trainers

The trainers were local and trained through classroom and fieldwork, and were key to the programme’s acceptability. Participants appreciated their understanding of local dynamics, respectful manner and relatability: “*She was a good listener and gave real-life examples. That made her acceptable to our family. We had a loving relationship.*” (FGD-Mother-in-law).

Many participants saw the trainers as sisters or friends. One young woman referred to her trainer as an angel: “*She was like an angel sent by God. I used to feel hesitant with my in-laws, she gave me the confidence and now I can talk to them freely.*” (IDI-Nadia, aged 20 years).

Trainers also established trust with other family members. One participant shared how her presence uplifted the household mood: “*Everyone used to ask when she would come. Even my mother-in-law felt better after talking to* her.” (IDI-Sana, aged 22 years).

##### Subtheme 2.4: Perceived positive impact of the intervention

Participants frequently reported improved psychosocial well-being and readiness for parenting. They felt enhanced self-confidence, assertiveness and communication, which improved their interpersonal relationships. Two women who initially had strained marriages described significant improvement: “*Before the programme, my relation with my husband was not good. Now, better communication has strengthened it.*” *.…every time I wanted to talk to my husband, I reminded myself what Baji (trainer) said and it worked.*” (IDI-Zara, aged 19 years).

Husbands also observed positive changes. One noted his wife’s improved anger management, time management and self-care: “*She manages the home better… she takes her meals on time, drinks milk… I give her full marks in self-care.*” (IDI-Ali, aged 27 years).

Trainers also noted behaviour change. One recalled a participant who began consulting her mother-in-law before making decisions, leading to improved family dynamics: “*She (mother-in-law) said her daughter-in-law never consulted her before going to her maternal home… now she does, and that made her feel good and respond* positively.” (FGD- Trainers).

The programme helped participants feel more prepared for their parenting roles. A pregnant woman described becoming more mindful of her health during pregnancy: “*I was careless about my pregnancy, but after learning from Baji (trainer) that my health is important for my baby’s health, I started looking after myself.*” (FGD-Faiza, aged 21 years).

Another participant with a few-month-old baby felt that her improved communication and problem-solving skills enabled her to fulfil her role as a mother more confidently: “*Yesterday my daughter was unwell…. So first I asked my husband to take her to the doctor. Upon his refusal, I asked my neighbour to accompany me, and we went to the clinic by bus.*” (IDI-Sara, aged 21 years).

A husband who was actively engaged in the intervention shared how the parenting information supported his role: “*It helped me realise why my input is important, so I started taking better care of both my wife and child.*” (IDI-Hassan, aged 28 years).

#### Theme 3: Barriers to the intervention delivery

##### Sub-theme 3.1: Limited husband participation

Most husbands were unable to attend sessions due to work commitments or living away from home. A mother-in-law explained: “*My son couldn’t attend the programme due to his job. He comes home late, but I allowed the trainer to talk to my family.*” (FGD- Mother-in-law).

Cultural norms around gender segregation also discouraged male involvement: “*Men avoid sitting in female gatherings. It’s not our norm. They feel shy, lack confidence, and find it difficult to talk to women.*” (FGD- Sister-in-law).

In some cases, husbands showed a clear lack of interest, either avoiding the trainer or dismissing the intervention’s relevance. A wife complained about her husband saying: “*It was hard to share anything with my husband. He didn’t listen to such things and only did what he wanted.*” (IDI-Sara, aged 21 years).

##### Sub-theme 3.2: Family control and resistance

While some families were supportive (see Theme 2.1), others acted as gatekeepers, restricting access to the intervention. One participant who dropped out shared how her family discouraged her: “*All the family members asked me to leave the programme. They were angry and said, ‘Why does she (trainer) come when you’re busiest with housework? Tell her not to come.’*” (IDI-Noor, aged 20 years).

Fear of disclosing personal matters to outsiders also led to non-participation: “*It’s difficult for all families to take part. They (in-laws) don’t like outsiders coming to their homes or their daughters-in-law talking to them. They fear disclosure of private family matters*” (IDI*-*Safiya, mother-in-law*).*

A trainer described how dominant family members prevented a participant from engaging openly: “*The mother-in-law and sister-in-law were very domineering. The participant could never speak freely. Her sister-in-law would stop her, and she was afraid to talk in front of her mother-in-law. I knew not involving the family would be better, but without their involvement, I had no chance*” (FGD-Trainers).

Cultural constraints also affected implementation. Fear of offending in-laws and resistance to change limited some participants’ application of skills: “*It was difficult to practice the skills… my in-laws have an old thinking* style.” (IDI-Zara, aged 19 years).

Trainers highlighted similar concerns, especially around discussions on decision-making and family planning: “*Some families misinterpreted it as promoting contraception… they reacted like we were Lady Health* Workers.” (FGD-Trainer).

Lastly, external influence also played a role, as in the case of a participant whose husband was swayed by negative comments from others: “*Someone told my husband this programme is useless and questioned why he let me attend. He got angry and asked me to quit.*” (IDI-Ayesha, aged 19 years).

##### Sub-theme 3.3: Competing responsibilities and perceived time burden

Excessive household or agricultural work made it difficult for some women to participate fully. The intervention comprised 10 core sessions containing key messages and skills that were essential for intervention completion. These were supplemented by booster sessions, which were desirable but not mandatory. Some participants and trainers reported that the number of sessions was sometimes perceived as excessive, particularly the follow-up sessions, leading some women to prefer receiving only the core sessions. As one trainer noted: “*They weren’t willing to attend follow-ups (due to time constraints). They used to say, ‘Please come once and do everything in one (core) session.’ I ended up doing that with most of them.*” (FGD-Trainer).

Another participant, who was required to support her husband in the fields during the harvesting season, was unable to continue receiving the sessions: She explained, “*When Baji (trainer) came, I couldn’t spare time. Everyone was busy harvesting, and I had to manage the* house.” (IDI-Noor, aged 20 years).

##### Subtheme 3.4: Internal barriers

While the barriers mentioned above were mainly external barriers, some participants also reported internal barriers such as low literacy and lack of motivation to engage with the intervention, especially during early sessions. Participants with low literacy levels experienced early challenges in understanding materials and completing workbook activities, as reported by a participant with only 5 years of schooling: “*Initially it was difficult… with time I understood better, but at times I do struggle to remember or write down activities.*” (IDI-Ayesha, aged 19 years).

Some participants found certain techniques, like breathing exercises, hard to practice at first, but improved with support, “*It was difficult to do breathing exercises. But with help from Baji (trainer), I got better.” (Zara, aged 19 years).*

Lack of motivation in some cases further hindered outcomes. One husband noted: “*My wife didn’t take advantage of the programme. It was her responsibility to act on what she learned, but she seemed disinterested*” (IDI-Hassan, aged 28 years).

#### Theme 4: Recommendations for programme improvement

##### Sub-theme 4.1: Leveraging technology to engage men

As noted in Theme 3.1, husbands’ participation in the programme was limited due to work commitments, physical absence and cultural norms. Participants suggested that using technology could help deliver key messages to men in a more accessible way. Most husbands owned smartphones and were active on social media platforms such as WhatsApp and Facebook. Sending audio or text messages was considered a practical method to share life skills content: “*These days everybody uses smartphones. People don’t read books anymore; they get their information online. It would be useful to upload this programme on the internet or send messages through* WhatsApp.” (IDI-Sara, aged 21 years).

Others recommended using audio recordings of sessions to allow husbands to listen at their convenience: “*They can attend the session online, or if not free, listen to an audio recording whenever they have* time.” (FGD-sister-in-law).

One participant proposed adapting the intervention into a television drama series or integrating it into morning shows, noting that families commonly watch these together: “*Show people this programme as a drama. People watch dramas on TV and mobile* phones.” (FGD-sister-in-law).

##### Sub-theme 4.2: Engaging men through male-focused delivery channels

Given cultural restrictions on gender mixing, several participants recommended training male delivery agents to work with male participants. This would help engage men who are reluctant to interact with female trainers: “*Send men to talk to men. Make a programme for men and have it delivered by men. My husband would not sit with a female trainer. They not to intrude where women are* meeting.” (IDI-Sana, aged 22 years).

Participants also suggested involving trusted community figures such as LHWs, retired teachers, or hospital staff who already interact with young couples or get intervention delivered at facilities: “*Mostly newly married couples visit hospitals for (medical) advice. This intervention could be delivered* there.” (FGD-Trainer).

##### Subtheme 4.3: Reducing and restructuring sessions

Some participants recommended delivering the intervention in group settings to increase efficiency and engagement. Preferred venues included schools, local centres or hospitals. Group sessions were seen as a way to overcome household constraints and reach multiple women at once: “*Sessions can be held in groups at a nearby school. Women can come for 1 - 2 hours. People have transport and wouldn’t face difficulty* attending.” (IDI-Hassan, aged 28 years).

*Similar views were echoed by other family member, who further suggested involving religious leaders to advocate participation in such programmes: “Create centres in different areas. Announce through mosques. Pick a day when kids are at school so mothers can attend* easily.” (FGD-Sister-in-law).

To address concerns about time and workload, many suggested reducing the number of sessions, eliminating follow-ups, or shifting from weekly to fortnightly delivery: “*The number of sessions should be reduced. Hold sessions every 15 days. The trainer can revise the previous session and ask whether participants applied the skills*” (IDI-Hassan, aged 28 years).

Finally, there were recommendations to improve the workbook by simplifying instructions, incorporating pictorial illustrations for non-literate users and highlighting key points: “*The workbook can be improved by adding clearer instructions or pictures to help participants understand the* activities.” (IDI-Fatima, aged 19 years).

## Discussion

This study explored the experiences of participants, their families and delivery agents involved in the Preparing for Parenthood (PPP) life skills programme for married adolescent women, their spouses and families in rural Pakistan. The findings from the qualitative study indicated that the PPP was well accepted and perceived as highly relevant. Participants appreciated the home-based delivery and applied the life skills learned, which helped improve their personal wellbeing, interpersonal relationships and readiness for parenting. Family involvement and the acceptability of trainers were key to sustained engagement. While most husbands did not attend sessions, many received information through their wives. Missed sessions were largely due to limited family support or heavy domestic workloads. Participants suggested using technology to engage men and expand the intervention’s reach.

The data showed benefits that align with existing evidence that life skills training can enhance self-awareness, emotional regulation, communication and decision-making (Singla et al., 2020). In this study, such improvements contributed to greater confidence and competence in parenting. Many adolescent women reported feeling more prepared to care for their children, manage household responsibilities and navigate family dynamics – suggesting that the PPP intervention equipped them not only with interpersonal and cognitive competencies but also with a sense of agency in their new parenting roles. These findings indicate that beyond foundational skills, the intervention catalysed broader socioemotional development, an often-overlooked area in adolescent programmes in South Asia (Nasheeda et al., 2019).

A key strength of the PPP intervention was its delivery model, which likely played a critical role in enhancing participants’ perceived psychosocial outcomes and readiness for parenthood. The use of LHWs for participant recruitment, coupled with the delivery of home-based sessions by trained local women, proved instrumental in fostering consistent engagement. Local trainers were valued for their linguistic competence and their psychosocial understanding of the needs of the target population. This model aligns with evidence supporting the effectiveness of delivering psychosocial interventions through peers and LHWs in LMICs (Fuhr et al., 2014; Atif et al., 2016).

While the intervention included structured content to ensure consistent delivery of core components (e.g., key health messages and skill-based techniques), a degree of flexibility was necessary to enable trainers to adapt communication styles, language and examples to the local context. Such adaptations were particularly important for enhancing comprehension and engagement. Importantly, the approach facilitated trust-building and culturally responsive care – particularly valuable in gender-restrictive contexts where adolescent girls often face limited mobility and autonomy (Barnett et al., 2023).

Moreover, the intervention incorporated ‘common elements’ that align with several evidence-based CBT approaches previously tested in LMICs (Singla et al., 2017). As outlined in the PPP intervention development paper (Atif et al., 2021), the programme drew on techniques and strategies from the WHO adopted *Thinking Healthy Programme* – a community-based psychosocial intervention developed and tested in Pakistan – which includes CBT strategies such as thought challenging, behaviour activation and problem solving, and family engagement (Rahman et al., 2008). The integration of such evidence-based components contributed to participants’ reported improvements in self-care, interpersonal relationships and parenting confidence. These outcomes underscore the potential of community-delivered, home-based psychosocial interventions to empower young couples in their transition to parenthood.

The study highlights that the inclusion of family members was particularly important, as they function as key gatekeepers who can either enable or restrict adolescents’ access to information, support and behavioural change. While some facilitated participation, others restricted it, reflecting the entrenched gender and generational hierarchies in South Asian households (Jejeebhoy and Sathar, 2001; Choudhry et al., 2019). Consistent with this, evidence from non-Western settings, including interventions in West Africa addressing harmful practices such as child marriage and female genital mutilation, suggests that older family members, such as grandmothers, can at times exert a negative influence; however, their advisory and caregiving roles are often beneficial (Aubel, 2024) and they can adopt new knowledge to positively shape maternal and child health practices (Aubel et al., 2004). Similarly, in Pakistan, the Thinking Healthy Programme engaged older family members such as parents-in-law by emphasising shared goals around maternal wellbeing and optimal child development, thereby fostering their support for behaviour change (Rahman et al., 2008). Taken together, these findings underscore how intra-household power dynamics can both enable and constrain adolescents’ engagement and agency. Interventions should therefore engage key family elders and gatekeepers through highlighting common agendas, while preserving adolescents’ autonomy, and may benefit from more explicitly addressing household power structures.

The PPP intervention approached sensitive issues such as limited women’s empowerment, rights violations and domestic abuse indirectly, by promoting care, respect and a supportive family environment for child-rearing. While participants reported improvements in interpersonal relationships, problem-solving and decision-making, the absence of explicit discussion of these issues suggests potential limitations in addressing deeper power dynamics. These challenges were further compounded by women’s substantial domestic responsibilities, including household chores and seasonal agricultural work, echoing wider evidence on how unpaid responsibilities restrict engagement in development programmes (Ali et al., 2022). Together, these findings highlight the need for future interventions to more directly, yet safely, address gendered power structures, while also adopting flexible delivery approaches such as condensed sessions, mobile-based learning or community group models may help accommodate competing demands, reduce dropout and enhance programme reach (Beall et al., 2014; Cox et al., 2025), although such adaptations require careful cultural consideration to ensure acceptability and safety.

Low male participation emerged as a key challenge, driven by work demands, gender norms and limited interest, mirroring broader patterns of male disengagement in psychosocial interventions (Ali et al., 2022; Morgan et al., 2022). However, both male and female participants proposed practical solutions such as male facilitators, audio messages, mobile apps and media content like TV dramas. These suggestions highlight adolescents’ openness to hybrid and culturally resonant delivery formats, which could be leveraged to support joint parenting roles.

Overall, the findings suggest that the PPP intervention addressed an unmet need for structured socioemotional guidance during early marriage. As one participant put it, “*I was told to live happily with my husband and in-laws, without telling me how to*” illustrating the knowledge and support gap the intervention helped to bridge.

### Strengths and limitations

This study has several strengths. It focuses on married adolescent women, an underrepresented population in low-resource settings and is embedded within a feasibility randomised controlled trial, enhancing the contextual relevance of the findings. The inclusion of multiple perspectives, adolescents, spouses and family members, provided a comprehensive understanding of the social dynamics influencing engagement. Additionally, the use of locally trained facilitators and home-based delivery strengthens cultural appropriateness and the transferability of findings to similar settings.

However, some limitations should be considered. The participants were all from one rural District of Punjab, and their views might not be representative of the entire population. We purposively selected willing participants for the interview. Those who did not engage with the process might have divergent views, which would not be captured. Moreover, husbands and non-completers were underrepresented, with only two interviews conducted for each group. Lastly, social desirability bias may also have shaped participants’ responses.

### Implications for practice and policy

Findings from this study offer actionable lessons for scaling up life skills interventions in similar settings. First, integrating families, particularly influential in-laws, into the programme design can promote support and retention. Second, gender-sensitive adaptations should go beyond engaging men as co-parents to address intra-household power dynamics, including women’s restricted decision-making and potential exposure to violence, through contextually appropriate and culturally sensitive strategies. Third, investing in delivery agents’ relational and cultural skills enhances trust and programme uptake. Overall, interventions must be tailored to address both individual and systemic barriers is key to equipping young couples with the skills and confidence to fulfil their parenting roles.

## Conclusion

Overall, the PPP intervention was experienced as relevant, empowering, and impactful by most participants. By building core life skills, the programme helped married adolescent women feel more confident and capable in their parenting roles. Ensuring gender-sensitive delivery, addressing family power dynamics and offering flexible formats will be crucial for sustaining and scaling such interventions in resource-constrained, patriarchal contexts.

## Supporting information

10.1017/gmh.2026.10240.sm001 Atif et al. supplementary material 1 Atif et al. supplementary material

10.1017/gmh.2026.10240.sm002 Atif et al. supplementary material 2 Atif et al. supplementary material

## Data Availability

The data that support the findings of this study are not publicly available due to participant confidentiality restrictions but are available from the corresponding author upon reasonable request.

## List of abbreviations

CBT: Cognitive Behavioural Therapy
COREQ: Consolidated Criteria for Reporting Qualitative Research
DALYs: Disability-Adjusted Life Years
FGDs: Focus Group Discussions
HDRF: Human Development Research Foundation
IDIs: In-depth Interviews
LHWs: Lady Health Workers
LMICs: Low- and middle-income countries
NIPS: National Institute of Population Studies
PPP: Preparing for Parenthood
UNICEF: United Nations Children’s Fund
WHO: World Health Organisation

## Supplementary material

The supplementary material for this article can be found at http://doi.org/10.1017/gmh.2026.10240.

## Long descriptions

### Table 1. Long description

The table is titled Session 0: Introduction to the program. It consists of two columns: Title of core sessions 1 to 10 and Major focus of session.

* Session 1: Knowing and appreciating each other focuses on Family support and communication skills.

* Session 2: Looking after yourself focuses on Thought challenging and behavioural activation.

* Session 3: Looking after yourself and others around you focuses on Thought challenging and behavioural activation.

* Session 4: Listening and understanding each other focuses on Communication skills and awareness.

* Session 5: Communicating respectfully and positively focuses on Communication skills and assertiveness.

* Session 6: Seeking and valuing each other’s opinion focuses on Communication skills and decision making.

* Session 7: Preparing for parenthood: pre-pregnancy focuses on Awareness and education of pre-pregnancy care, behavioural activation, and problem solving.

* Session 8: Preparing for parenthood: during-pregnancy focuses on Awareness and education of care during pregnancy, behavioural activation, and problem solving.

* Session 9: Preparing for parenthood: post-pregnancy focuses on Awareness and education of post pregnancy case, behavioural activation, and problem solving.

* Session 10: Managing problems focuses on Problem solving.

A note at the bottom states that each core session has a follow-up session delivered the following week, totaling 20 sessions.

Navigate back to Table 1..

### Table 2. Long description

The table contains seven columns: Reference Number, Pseudonym, Age, Number of married years, Family structure, Years of schooling, and Number of children.

* Reference C 1: Amina, 21 years old, married 1 year, Joint family, 10 years schooling, Nil children.

* Reference C 2: Sana, 22 years old, married 1 year, Joint family, 12 years schooling, Nil children.

* Reference C 3: Nadia, 20 years old, married 11 months, Joint family, 10 years schooling, Nil children.

* Reference C 4: Zara, 19 years old, married 1 year, Joint family, 12 years schooling, Nil children.

* Reference C 5: Fatima, 19 years old, married 10 months, Joint family, 10 years schooling, Nil children.

* Reference C 6: Ayesha, 19 years old, married 10 months, Joint family, 5 years schooling, Nil children.

* Reference C 7: Sara, 21 years old, married 1 year, Joint family, 9 years schooling, 1 child.

* Reference C 8: Saba, 20 years old, married 2 years, Joint family, 12 years schooling, Nil children.

* Reference N C 1: Nabila, 20 years old, married 1 year 3 months, Joint family, 10 years schooling, Nil children.

* Reference N C 2: Maira, 20 years old, married 2 years, Joint family, 10 years schooling, 1 child.

* Reference H C 1: Hassan, 28 years old, married 10 months, Joint family, 5th year schooling, Nil children.

* Reference H N C 2: Ali, 27 years old, married 2 years, Joint family, 8 years schooling, 1 child.

Footnotes indicate C equals participants completing the intervention (at least 10 sessions), N C equals non-completers, H C 1 is the husband of a completer, and H N C 1 is the husband of a non-completer.

Navigate back to Table 2..

### Table 3. Long description

The table consists of three columns: References, Number of participants, and Relationship to the study participants.

* Row 1: Reference F 1, 3 participants, Family: Mother-in-law, sister-in-law, participant.

* Row 2: Reference F 2, 4 participants, Family: Mother-in-law, sister-in-law times 2, participant.

* Row 3: Reference F 3, 5 participants, Family: Mother-in-law, sister-in-law times 3, participant.

* Row 4: Reference T and F, 7 participants, Trainers and Field Coordinator.

Navigate back to Table 3..

### Table 4. Long description

The table is organized into three columns: Themes, Sub-themes, and Key findings.

Theme 1.0: Perception of the intervention.

Sub-themes include 1.1 Expectations and motivation, 1.2 Satisfaction with delivery format, 1.3 Techniques and materials, and 1.4 Ease of application of life skills.

Key findings: Participants welcomed the intervention for life skills and parenting prep, appreciating the flexible home-based format and interactive storytelling. They reported applying skills in self-care and stress management.

Theme 2.0: Facilitators of intervention engagement and delivery.

Sub-themes include 2.1 Family support and participation, 2.2 Sharing knowledge with husbands and in-laws, 2.3 Trust and rapport with trainers, and 2.4 Perceived positive impact.

Key findings: Family and in-law support were critical. Trust in locally recruited, culturally attuned trainers sustained involvement. Improved emotional well-being and decision-making were noted.

Theme 3.0: Barriers to intervention engagement and delivery.

Sub-themes include 3.1 Limited husband participation, 3.2 Family control and resistance, 3.3 Competing responsibilities and time burden, and 3.4 Internal barriers.

Key findings: Barriers included husbands’ work demands, cultural norms, family resistance, domestic/agricultural duties, and low literacy.

Theme 4.0: Recommendations for programme improvement.

Sub-themes include 4.1 Leveraging technology to engage men, 4.2 Engaging men through male-focused delivery channels, and 4.3 Reducing and restructuring sessions.

Key findings: Suggestions include using W h a t s A p p or television to reach men, using male-led delivery channels, and moving to group formats at community venues like schools to improve efficiency.

Navigate back to Table 4..
