# Supplementary material for: “I was told to live happily… without telling me how”: Lived experiences of married adolescents, their spouses and families with a life skills intervention in rural Pakistan – A qualitative study
Source: Glob Ment Health (Camb). 2026 Jun 9;13:e162. doi: 10.1017/gmh.2026.10240 (PMC13433332; doi:10.1017/gmh.2026.10240)
Supplement: Supplementary file 2 [file S2054425126102404sup001.docx]

**Appendix 1: Topic Guide for the married adolescent females who received the intervention**

- ***Exploring wives’ expectations from the PPP***
  - What made you decide to participate?
  - What were your expectations from receiving the PPP?
  - How far were these expectations met?
- ***Exploring wives’ experience of receiving the PPP***
  - What are your views about the number and duration of the sessions?
    - What are your views about the follow-ups, were they useful/ not useful?
  - What are your views about the format of delivery?
    - How it could be addressed?
  - How acceptable/unacceptable was the PPP material?
  - How comprehensible/incomprehensible was the PPP material?
  - Was the PPP material enough to address all your issues?
  - Should the family members be welcomed during the intervention delivery?
    - If yes how it will benefit
    - If no why
  - What are your views about different methods used in PPP?
    - Stories
      - What are your views of the stories?
      - How relevant were the characters in the stories to you?
    - Activities
      - Were you able to engage your husband in the activities?
    - Workbook
      - How doable were the activities outlined in the workbook?
      - Did you experience any barriers in doing the homework?
    - Illustrations
    - Others
  - Which aspects of PPP did you find least useful…? why?
    - How they can be improved?
- ***Exploring the perceived impact of receiving PPP on the wives***
  - What was the impact of receiving the PPP on you – both GOOD and BAD?
  - How much and how you have applied the skills taught in PPP in your day to day lives.
  - What impact did PPP have on you (both good and bad):
    - Communication skills
    - Confidence
    - Self-care
    - Relationship with significant others
    - Preparing for parenthood
    - Others
  - Did it motivate you to attend the LHWs’ group?
  - Has the information been passed on to others?
  - Do you think receiving the intervention would have short-term or long-term impact on you? Please explain.
- How the programme can be improved?
- If we want the programme to reach out to young couples all over Pakistan how this can be done?
- ***Exploring barriers in receiving the PPP***
  - Did you experience any barriers in receiving the PPP?
    - If yes what were those barriers?
    - How they were overcome?
  - Did you experience any challenges in using the life skills learnt during the programme?
  - What were those challenges?
    - How were those challenges overcome?
  - Did significant members of your family such as husbands participated in receiving the intervention?
    - What stopped them from part?
    - How can their involvement be improved?
  - Can use of technology help in improving the involvement of husbands?
- ***Exploring wives and their families’ views towards PPP trainers***
  - How acceptable/unacceptable was the PPP trainers for you and family?
    - Who else could have delivered the PPP?
  - In your opinion how effective/ineffective were PPP trainers in delivering the PPP and why?
  - What personal characteristics of PPP trainers were most favored by you and your family?
  - What personal characteristics of PPP trainers were least favored by you and your family?
  - How did you and your family feel meeting PPP trainers on a regular basis and why?
  - How was your and your family’s behaviour (cooperative/ un-cooperative) towards PPP trainers?
    - What were the reasons for your attitude/behaviour?

**Topic Guide for the married adolescent females who dropped-out**

- ***Exploring wives’ reasons for dropping out of the programme***
  - What made you decide to participate at the first place?
    - Was the programme different from what you expected?
  - What are your views about the methods of delivery?
    - How did you find the methods of delivery?
      - Appropriate/inappropriate
        - Format
        - Number and duration of sessions
  - What are your views about the programme material?
    - Acceptable/unacceptable to you and family
    - Comprehensible/incomprehensible to you and family
    - Relevant/irrelevant
    - Was the PPP material enough to address all your issues?
  - What are your views about the impact of the programme?
    - Did you manage to apply the skills taught in the sessions you received in your day to day lives.
    - Did the programme have any impact on you – both good and bad?
      - Communication skills
      - Confidence
      - Self-care
      - Relationship with significant others
      - Preparing for parenthood
      - Others
    - Did it motivate you to attend the LHWs’ group?
  - What are your views about the PPP trainers?
    - Acceptable/unacceptable.
    - Effective/ineffective were PPP trainers in delivering the PPP and why?
    - Who else could have delivered the PPP?
  - What barriers did you experience in receiving the PPP?
    - From your husband, parents-in-law
    - Work commitments
  - What made you decide to leave the programme?
    - Was it your decision?
    - Were you being forced by your family?
    - Would you have continued if allowed to do so?
  - What would have motivated you to continue with the programme?
    - Less sessions, Change of venue, Different trainers, Use of technology

**Topic Guide for Husbands who received at least one session**

Your wife was receiving the PPP weekly sessions for the last six months. You were invited by our PPP trainers to receive these sessions with her.

- ***Exploring husband’s experience of receiving the PPP***
  - Did you manage to receive any of the PPP sessions with your wife?
  - How was your experience of receiving the session/s?
  - What are your views about the number and duration of sessions?
  - What are your views about the follow-ups, were they useful/ not useful?
  - What are your views about the format of delivery?
  - How acceptable/unacceptable was the PPP material to you and your family?
  - How comprehensible/incomprehensible was the PPP material?
  - Was the PPP material enough to address all your issues?
  - Should the family members be welcomed during the intervention delivery?
    - If yes how it will benefit
    - If no why
  - What are your views about different methods used in PPP?
    - Stories
      - What are your views of the stories?
      - How relevant were the characters in the stories to you?
    - Activities
      - Were you able to engage your husband in the activities?
    - Workbook
      - How doable were the activities outlined in the workbook?
      - Did you experience any barriers in doing the homework?
    - Illustrations
    - Others
  - Which aspects of PPP did you find least useful…? why?
    - How they can be improved?
- ***Exploring husband’s perceived impact of receiving PPP (both good and bad)***
  - Did you find the PPP intervention useful? If not why?
  - How much you or your wife has applied the skills taught in PPP in your day to day lives.
  - What impact did PPP have on you or your wife (both good and bad)?
    - Communication skills
    - Confidence
    - Self-care
    - Relationship with significant others
    - Preparing for parenthood
  - Did it motivate your wife to attend the LHWs’ group?
  - Has the information been passed on to others?
  - Do you think receiving the intervention would have short-term or long-term impact on you or your wife? Please explain.
- How the programme can be improved?
- If we want the programme to reach out to young couples all over Pakistan how this can be done?
- ***Exploring barriers in receiving the PPP***
  - Did you or your wife experience any barriers in receiving the PPP?
    - If yes what were those barriers?
    - How they were overcome?
  - Did you or your wife experience any challenges in using the life skills learnt during the programme?
  - What were those challenges?
    - How were those challenges overcome?
  - Did significant members of your family such as your mother or father participated in receiving the intervention?
    - What stopped them from part?
    - How can their involvement be improved?
  - Can use of technology help in improving the involvement of your family members?
- ***Exploring husbands and their families’ views towards PPP trainers***
  - How acceptable/unacceptable was the PPP trainers for you and family?
    - Who else could have delivered the PPP?
  - In your opinion how effective/ineffective were PPP trainers in delivering the PPP and why?
  - What personal characteristics of PPP trainers were most favored by you and your family?
  - What personal characteristics of PPP trainers were least favored by you and your family?
  - How did you and your family feel meeting PPP trainers on a regular basis and why?
  - How was your and your family’s behaviour (cooperative/ un-cooperative) towards PPP trainers?
    - What were the reasons for your attitude/behaviour?

**Topic Guide for Husbands who didn’t receive any session**

You have not been able to receive the PPP programme. However, we do hope that your wife would have been sharing the PPP information with you and discussing the life skills learned during the sessions. Based on the information you have received we would like to ask you some questions.

- ***Exploring husband’s views about the programme***
  - *Did your wife discuss the life skills she learnt during the sessions with you?*
    - *If yes*
      - *Did you try to implement these skills in your day-to-day lives?*
    - *If no*
      - *What stopped her from discussing them with you*

- ***Exploring husband’s view about the impact of the programme on his wife***
  - *Have you noticed any change in your wife’s behavior/attitude?*
  - *If yes, what?*
    - *Self-care, Interpersonal relationship, Assertiveness, Confidence, communication skills …….*
- ***Exploring husband’s views for not attending the PPP sessions***
  - *What stopped you from attending the sessions.*
    - *Lack of interest*
    - *Work commitments*
    - *Away from home (If you would have been home, would you have taken part in it?)*
    - *Did not like interference in their personal lives*
    - *Parents doesn’t allow*
    - *Others*
- ***Exploring views on improving husbands’ involvement in the programme***
  - *How do you thing husband’s involvement can be improved?*
  - *Can TECHNOLOGY play any role in it?*
    - *SMS*
    - *App*

**Topic Guide for the PPP trainers**

- ***Exploring trainers’ experience of receiving the PPP training***
  - How was your experience of receiving the training?
    - Venue of training
    - Duration of training
    - Amount of material covered on
    - Techniques used to deliver information
      - Presentation
      - Lectures
      - role plays
  - How useful/not useful was the information for the actual delivery of the PPP?
    - How it could be improved?
  - How did you find the field training?
  - Did the training equip you for this role? If yes in which ways? If not why?
    - How could PPP training be improved?
- ***Exploring PPP trainers’ experience of delivering the sessions***
  - What are your views about the number and duration of the sessions?
    - What are your views about the follow-ups, were they useful/ not useful?
  - What are your views about the format of delivery?
    - How it could be addressed?
  - How acceptable/unacceptable was the PPP material?
  - How comprehensible/incomprehensible was the PPP information?
  - What are your views about different methods used in PPP?
    - Stories
      - What are your views of the stories?
      - How relevant were the characters in the stories to your participants?
    - Activities
      - Were you able to engage their husband in the activities?
    - Workbook
      - How doable were the activities outlined in the workbook?
    - What barriers did your participants experiences in doing their homework?
    - Others
  - Which aspects of PPP did you find least useful….why?
    - How they can be improved?
- ***Exploring the PPP trainers’ perceived impact of PPP on wives (both good and bad)***
  - Did wives find the PPP information useful if yes how? ……. if not why?
    - How it could be improved?
  - How practical/doable was the information given to wives?
  - What impact did PPP have on them (both good and bad):
    - Communication skills
    - Confidence
    - Self-care
    - Relationship with significant others
    - Preparing for parenthood
    - Others
  - Did it motivate them to attend the LHWs’ group?
  - Has the information been passed on to others?
  - Do you think receiving the intervention would have short-term or long-term on them? Please explain.
- How the programme can be improved?
- If we want the programme to reach out to young couples all over Pakistan how this can be done?
- ***Exploring barriers in delivering the PPP***
  - Did you experience any barriers in delivering the PPP?
    - If yes what were those barriers?
    - How they were overcome?
  - Did they experience any challenges in using the life skills learnt during the programme?
  - What were those challenges?
    - How were those challenges overcome?
  - Did significant members of their family such as husbands participated in receiving the intervention?
    - What stopped them from part?
    - How can their involvement be improved?
  - Can use of technology help in improving the involvement of husbands?
- ***Exploring PPP trainers experience of receiving supervision***
  - What is your experience of receiving supervision and how it could be improved?
    - How often they were held?
    - Which aspects of supervision were most helpful?
    - Did you get opportunity to freely express your concerns during supervisions? If yes how? If not why?
    - Which aspects of supervision were least unhelpful? Why and how could they be improved
- ***Exploring the impact of PPP on the trainers***
  - Did you use any of the life skills suggested in the programme?
  - What was the impact of using these life skills on your?
    - Communication skills
    - Confidence
    - Self-care
    - Relationship with significant others
    - Preparing for parenthood
    - Others
  - Did you experience any adverse effects of delivering PPP?

**Topic Guide for the family-in-law**

Your daughter-in-law/sister-in-law was receiving the PPP weekly sessions for the last six months. You were invited by our PPP trainers to receive these sessions with her.

- **Exploring family-in-law’s views about the PPP**
  - Did you manage to receive any of the PPP sessions?
  - **If NOT**
    - what stopped you from receiving the sessions?
    - Did you stop your daughter-in-law from receiving the programme? If yes why?
      - What were your worst apprehensions in relation to the programme?
        - Might corrupt the daughter-in-law, waste of time, fear of discloser of information
    - Did your son receive the sessions?
    - What stopped him from taking part?
    - How can his involvement be improved?
    - Can use of technology will help in improving his involvement or the involvement of other family members?
  - Did you manage to receive any of the PPP sessions?
  - **If YES**
  - How was your experience of receiving the session/s
  - What are your views about the number and duration of sessions?
    - What are your views about the follow-ups, were they useful/ not useful?
  - What are your views about the format of delivery?
  - How acceptable/unacceptable was the PPP material for your family?
  - How comprehensible/incomprehensible was the PPP material?
  - Was the PPP material useful to address some of your family issues?
  - What are your views about different methods used in PPP?
    - Stories
      - What are your views of the stories?
      - How relevant were the characters in the stories to you?
    - Activities
      - Were you able to engage your husband in the activities?
    - Workbook
      - How doable were the activities outlined in the workbook?
      - Did you experience any barriers in doing the homework?
    - Illustrations
    - Others
  - Which aspects of PPP did you find least useful…? why?
    - How they can be improved?
- ***Exploring family’s perceived impact of receiving PPP (both good and bad)***
  - What are the family’s views about the usefulness of the PPP?
  - How much your family has applied the skills taught in PPP in their day to day lives.
    - Can you give examples such as: Communication skills, Confidence, Self-care, Relationship with significant others, Preparing for Parenthood.
  - Did you find the PPP intervention useful for the young couple? If yes how? If not why?
  - Did it motivate your daughter-in-law to attend the LHWs’ group?
  - Has the information been passed on to others?
  - Do you think receiving the intervention would have short-term or long-term impact on the family? Please explain.
  - How the programme can be improved?
  - If we want the programme to reach out to young couples all over Pakistan how this can be done? For example, through technology
- ***Exploring family’ views towards PPP trainers***
  - How acceptable/unacceptable was the PPP trainers for the family?
    - Who else could have delivered the PPP?
  - In your opinion how effective/ineffective were PPP trainers in delivering the PPP and why?
  - What personal characteristics of PPP trainers were most favored by the family?
  - What personal characteristics of PPP trainers were least favored by the family?
  - How did the family feel meeting PPP trainers on a regular basis and why?
  - How was the family’s behavior (cooperative/ un-cooperative) towards PPP trainers?
    - What were the reasons for your attitude/behavior?
