# Supplementary material for: “I was told to live happily… without telling me how”: Lived experiences of married adolescents, their spouses and families with a life skills intervention in rural Pakistan – A qualitative study
Source: Glob Ment Health (Camb). 2026 Jun 9;13:e162. doi: 10.1017/gmh.2026.10240 (PMC13433332; doi:10.1017/gmh.2026.10240)
Supplement: Peer review history

# Reviewer report: Review: “*I was told to live happily… without telling me how*”: Lived experiences of married adolescents, their spouses and families with a life skills intervention in rural Pakistan – A qualitative study — R0/PR2

**DOI:** 10.1017/gmh.2026.10240.pr2

Intervention supporting young couples in Pakistan is an extremely important area of research. Appropriate stakeholders were interviewed/ engaged. Though most of the Coreq reporting areas have been mentioned. It would be nice to add if these guidelines were followed.

# Reviewer report: Review: “*I was told to live happily… without telling me how*”: Lived experiences of married adolescents, their spouses and families with a life skills intervention in rural Pakistan – A qualitative study — R0/PR3

**DOI:** 10.1017/gmh.2026.10240.pr3

**Authors:** Rochelle Burgess

I would like to congratulate the authors on the production of an important manuscript, which focuses on an in important and under researched area - of early married adolescents. I have a series of suggestions for updates to the article that will strengthen it - my feeling is that the manuscript sits on the border between a minor and major revision, but the corrections are not insurmountable - just changes to improve clarity and widen reflections on key concerns in the field of early marriage and mental health/wellbeing as a whole. Please note, the page numbers below relate to the document pages(top left of the pdf given for review) not the manuscript pages.

Impact statement:

Page 3, line 3: is this not redundant to say married adolescents and their spouses? Can you clarify early on if you spoke to mostly married women or men? I also query the use of the lived experience here. My feeling is that ideally, it would be better to discuss the intervention first, then introduce whose lived experience you are referring to. Is this the lived experience of the intervention? Or their lived experience as married adolescents? or both? I know this is the impact statement but it is the first thing people may read so getting this right is important.

Introduction:

Pg 3, line 53: Your wording to identify the relationship between early marriage and the social contexts is an under estimation of the bidirectionality between youth unemployment, economic disadvantage and early marriage - which has been shown in other studies globally (see for example: Wahhaj, Z., 2022. The economics of early marriage: causes, consequences, and policy solutions. In Handbook of labor, human resources and population economics (pp. 1-26)). I would re-work this section to highlight this complexity a bit more.

page 3, line 30 - The introduction/discussion of background literature needs a bit more attention. You have not yet adequately introduced why the parental engagement is so important to an intervention like PPP in the context of early marriage. An expansion on the drivers of early marriage (and the introduction of forced marriage into this discussion) would help to clarify this particular need in this setting - or to rule it out if the pathway differs in Pakistan around forced marriages.

Methods:

Page, line 20: More detail is needed around your selection criteria. Why did you include number of years married? did you need a minimum number of years for participation in the intervention? why? Could this detail be provided? at a minimum in the table? Also, it is not clear how people were recruited into the main intervention - so the lack of this detail is what makes it important to mention here.

line 28: What languages did the researchers speak? How were they connected to the study overall? Were they hired specifically to do this piece or were they embedded in the study in another way? Were they local to the context or no?

line 32: Saturation of what? experiences? Perspectives on the intervention? Different types of data collected? This really needs clarity, as there are differing debates on how to engage with saturation in terms of sampling, as well as within analyses. See for example: Hennink, M. and Kaiser, B.N., 2022. Sample sizes for saturation in qualitative research: A systematic review of empirical tests. Social science & medicine, 292, p.114523.

Page 5, line 1: You jump from discussion of in-depth interviews, to semi-structured interviews - which one is it? They are not automatically the same. Also, does this mean that if they are different, in-depth were written in another language other than Urdu?

Data analysis;

Page 5. line 18: This Braun and Clarke method has been expanded in a way that calls the team to differentiate between how they manage the idea of reflexivity, and how they deal with theory. Can you clarify if this was fully inductive analysis? or if you had structured organised your analysis around some theory/framework.

The Intervention:

Page 6 line 5: Could a summary of the content for each session be provided in a table or appendix? it would be helpful to understand this detail. It should be in the manuscript itself, and not an appendix.

Results

Pg 6, line 26: Does this mean that two interviews were done with couples (2 people in the interview?)

line 30: are the focus groups composed of just family members for the same family? or are they groups of mixed families coming together? This is important

Page 7, line 2: You have made a choice to discuss all three samples together as one corpus - this suggests that you are doing a more directed type of analysis to try and understand experiences of the intervention in a broad way that speak to each other. I am not sure if i would agree with this particular choice - as each group would have a distinctive experience of the intervention and for a thematic analysis. I wonder if you could elaborate on this choice a bit, and perhaps later in the limitations, articulate any limitations this may have posed (if you think there are any)

Theme 1

Page 7, line 28- The use of ID codes like this always comes across as dehumanising - I could understand if you had a huge sample you were discussing, but I would consider using pseudonyms instead.

Pg 7, line 19: Did all participants express this aspiration? in Braun and Clarke there is also an interest in discussing divergences/diversity within a theme - so if this wasn’t universal it may be worth while to mention what other expectations and hopes were identified.

Pg 7, line 28: The quotes in theme 1.2 identify a particular dimension of context that is worth mentioning - the type of household structures that young married couples live in. This may need to come in the background/context - but it needs to be somewhere to anchor the value of the approach a bit more. Everyone would value flexibility in delivery - but this particular manifestation of flexibility really aligns with the demands of the context.

Page 8, line 16-18 - Again, this quote highlights why discussion of the wider context is so important here again. Why is this dialect important? Who speaks it? is it a minority or majority language?

Page 11, line 1:

This sub theme is not labelled correctly - you are talking about barriers to mobilising knowledge/learning - not implementation. In fact it is actually a blend of ideas about challenges to people using the knowledge, and also delivering the sessions. Which suggests the need to revisit this sub-theme overall as they are different processes.

Page 11: line 19

This sub-theme may benefit from a re-naming, to avoid confusion around the idea of facilitators (as people in an intervention). Perhaps Enabling or supporting factors for delivery?

Pg 12, line 11-13

This quote is a bit unclear, could you perhaps elaborate on what is meant with this quote as a piece of evidence? Are you trying to say that participants are sharing inaccurate information? Did you explore what this meant in this context? It seems as though working with vignettes would result in broad storytelling later down the line? What types of information delivered in the intervention requires detailed transfers, and what is open to more interpretation? Can any intervention really control this?

Brief questionnaire findings

Page 14, line 23

There is no section in your methods describing how you analysed this brief questionnaire. The below section should be moved to the methodology, so that you are discussing findings all in one go.

Alternatively, you may want to actually integrate the qual and quant discussion - rather than have these very small numbers attempt to stand on their own as discussions of impact.

Pg 16, line 19-20:

This is the first time detailed information about intervention content appears, and is too late. I would add it to the section earlier in the methods, along with a new table as mentioned in my earlier comments.

line 25: You may want to word the claim about integration of CET into the programme as contributing to improvement, it may have contributed - but with what you have presented in this analysis you cannot say for sure, so you want to phrase this slightly differently.

Page 16, Line 30-33:

You may want to elaborate in this section how other interventions have included familiy members in other settings. For example, there is significant work from western Africa about the involvement of grandmothers in interventions targeting young women linked to harmful practices around child marriage and FGM, which could be worth reflecting on here. Aubel, J., 2024. Grandmothers–a cultural resource for women and children’s health and well-being across the life cycle. Global health promotion, 31(2), pp.23-33.

Aubel, J., Touré, I. and Diagne, M., 2004. Senegalese grandmothers promote improved maternal and child nutrition practices: the guardians of tradition are not averse to change. Social science & medicine, 59(5), pp.945-959.

Page 17, line 16:

community group interventions also struggle with the problems you are highlighting key findings on the way that household power limits the actions of young women in your analysis - though you have not picked up on this here. Ultimately, you may want to acknowledge this, and reflect on how interventions can engage with power dynamics as part of their efforts. For example, in the what works to end violence against women programme, interventions in Uganda within relationships had clear discussions of power - is this needed? why or why not? how could it be approached in this particular context?

Line 19

Overall in your discussion, I didn’t see any reflection on violence as it relates to or was addressed in the intervention. Given that a few of the quotes under your theme of restricted access appear to allude to this - and your introductory framing highlighting high levels of IPV linked to early marriage made me feel as though it was a concern and part of the goals of the interventions. Did this not manifest? Could you elaborate a little on this point in the discussion?

Implications for practice and policy

Line 29-31 - This is not the only gender sensitive point to consider - see my earlier comments around violence and the restricted decision making power of women in households and communities as worth engaging with in this paper.

Once these points are addressed I feel that the manuscript will be strong enough for publication, and make a really critical contribution to our evidence base. I look forward to reading a revised version in due course.

# Editor report: Recommendation: “*I was told to live happily… without telling me how*”: Lived experiences of married adolescents, their spouses and families with a life skills intervention in rural Pakistan – A qualitative study — R0/PR4

**DOI:** 10.1017/gmh.2026.10240.pr4

**Affiliations:** Pravara Institute of Medical Sciences, India

No accompanying comment.

# Editor report: Decision: “*I was told to live happily… without telling me how*”: Lived experiences of married adolescents, their spouses and families with a life skills intervention in rural Pakistan – A qualitative study — R0/PR5

**DOI:** 10.1017/gmh.2026.10240.pr5

**Affiliations:** Johns Hopkins University Bloomberg School of Public Health, United States

No accompanying comment.

# Reviewer report: Review: “*I was told to live happily… without telling me how*”: Lived experiences of married adolescents, their spouses and families with a life skills intervention in rural Pakistan – A qualitative study — R1/PR7

**DOI:** 10.1017/gmh.2026.10240.pr7

**Authors:** Rochelle Burgess

Thank you for your responses to queries. I have nothing further to add and am happy for the manuscript to be published.

# Editor report: Recommendation: “*I was told to live happily… without telling me how*”: Lived experiences of married adolescents, their spouses and families with a life skills intervention in rural Pakistan – A qualitative study — R1/PR8

**DOI:** 10.1017/gmh.2026.10240.pr8

**Affiliations:** Pravara Institute of Medical Sciences, India

No accompanying comment.

# Editor report: Decision: “*I was told to live happily… without telling me how*”: Lived experiences of married adolescents, their spouses and families with a life skills intervention in rural Pakistan – A qualitative study — R1/PR9

**DOI:** 10.1017/gmh.2026.10240.pr9

**Affiliations:** Johns Hopkins University Bloomberg School of Public Health, United States

No accompanying comment.
